# GenoTox Cell Counter: A mobile App to standardize cell counting in genotoxicity testing

**DOI:** 10.1590/1678-4685-GMB-2025-0255

**Published:** 2026-08-24

**Authors:** Gumercindo Pimentel-Peralta, Yennifer Alfaro, Juliana Da Silva

**Affiliations:** 1 Universidade Federal do Rio Grande do Sul (UFRGS), Programa de Pós-Graduação em Genética e Biologia Molecular (PPGBM), Porto Alegre, RS, Brazil.; 2 Universidade La Salle, Laboratório de Genética Toxicológica, Canoas, RS, Brazil.; 3 Universidade Federal do Rio Grande do Sul (UFRGS), Programa de Pós-Graduação em Ciência e Tecnologia de Alimentos (PPGCTA), Porto Alegre, RS, Brazil.

**Keywords:** Genotoxicity, DNA damage, biomarkers, mobile application, cell counting

## Abstract

The analysis of genotoxicity assays has long posed a major challenge for the standardization of analyses in environmental and occupational settings, as well as in assessing health impacts across various diseases. In this context, we present a mobile application designed to facilitate and standardize scoring in micronucleus, comet, chromosomal aberration, and erythrocyte abnormality assays. The app offers a portable and intuitive interface that allows users to save, resume, and share counting sessions efficiently, supporting both laboratory and field settings. By digitizing the workflow, it enhances data organization, traceability, and time management while preserving the rigor of microscopic analysis. Its adoption offers a practical and reliable alternative to manual tallying, contributing to the evolution of genotoxicity testing practices through mobile technology.

In recent decades, mobile devices have profoundly transformed how information is accessed, managed, and shared across multiple disciplines, including healthcare and scientific research (Ventola, 2014; Aydin and Silahtaroglu, 2021; Istepanian, 2022). Their portability, connectivity, and processing power have enabled the development of specialized applications that optimize tasks previously confined to desktop environments or paper-based workflows. In the fields of cell biology and experimental toxicology, this technological evolution has opened new possibilities for improving the efficiency, traceability, and reproducibility of laboratory procedures (Zeng et al., 2018).

Genotoxicity assays are essential tools for detecting genetic damage induced by physical, chemical, or biological agents. Key methodologies include buccal and *in vitro* micronucleus (MNs) analysis, applied to human populations, animal groups, and cultured cell lines (Fenech et al., 1999; Thomas et al., 2009; Carracedo et al., 2025; Da Costa *et al*., 2025; Dai et al., 2025), the alkaline comet (AC) assay (Da Silva et al., 2000; Picinini-Zambelli et al., 2025), chromosomal aberration (CAA) evaluation (Clare, 2012; Deora et al., 2025), and studies using plant models like *Allium cepa* MN (MNAC) assay (Leme and Marin-Morales, 2009; Bonciu et al., 2018). A critical step in these protocols is the precise scoring of specific cellular structures, traditionally performed using tally sheets or mechanical counters. However, such approaches are prone to transcription errors, data loss, and limited integration with digital workflows, ultimately compromising data accuracy and reproducibility (Gupta et al., 2023).

Recent developments have demonstrated the feasibility of using smartphones for automated cell counting and viability analysis. For instance, Kang et al. (2019) designed a field-portable, smartphone-based cell counter capable of distinguishing live and dead cells with high precision, integrating hardware and software components for on-site detection. Their Android application, combined with a custom microscope, proved to be accurate, rapid, and cost-effective, highlighting the potential of mobile platforms in biological diagnostics and field testing. Similarly, Thurman et al. (2015) validated an application-based cell counter for clinical laboratory use, demonstrating excellent agreement with traditional analog devices across multiple cell types and users, and confirming that touchscreen interfaces do not compromise counting accuracy.

Beyond technical advances, the broader mobile health (mHealth) app ecosystem continues to expand rapidly. Aydin and Silahtaroglu (2021) analyzed over 1,000 mobile health applications and identified key factors influencing user adoption and satisfaction. Their findings indicate that features such as data privacy, feedback mechanisms, social sharing, and intuitive design are critical for user engagement and sustained use. These insights underscore the importance of usability, transparency, and accessibility in ensuring the effective integration of mobile tools into health-related workflows.

To address similar challenges in genotoxicity workflows, we developed the GenoTox Cell Counter (GCC), the first mobile platform specifically designed to streamline cell counting in genotoxicity assays. Beyond recording and sharing data securely, GCC integrates seamlessly into both laboratory and field workflows, offering an intuitive interface that supports researchers, trainees, and professionals alike. Its implementation across different genotoxicity protocols has improved organizational efficiency, enhanced data traceability, and reduced administrative workload, while maintaining the analytical rigor essential for microscopic evaluation. This article presents the development, technical design, and validation of GCC, highlighting its potential as a practical tool for modernizing genotoxicity testing.

The GCC mobile application was developed using the Flutter framework (SDK version 3.32.8, https://flutter.dev), an open-source UI toolkit from Google (Aung et al., 2024). Development was conducted in the Visual Studio Code v1.104.1 (https://code.visualstudio.com) using the Dart programming language (SDK version 3.8.1, https://dart.dev), Android Studio (Google LLC), on a Linux Ubuntu 24.04.3 LTS operating system (https://ubuntu.com/).

Flutter was selected for its single codebase architecture, which facilitates rapid development and ensures feature parity between Android and iOS platforms. Its stateful hot-reload feature further streamlined the UI design process. The application was compiled into native binaries for both operating systems, maximizing performance and ensuring compatibility with a wide range of devices commonly used in laboratories and field settings.

Key Flutter packages included provider for state management, shared_preferences v2.3.3 (https://pub.dev/packages/shared_preferences) for local data storage, and path_provider v2.1.5 (https://pub.dev/packages/path_provider) for file access. The app was tested using Flutter’s built-in testing framework, including widget tests and manual trials on physical devices and emulators. Android builds targeted SDK version 35 (minimum SDK 23), ensuring compatibility with devices running Android 5.0 and above, while iOS builds supported devices running iOS 12.0 or later.

All data recorded within the app is stored locally; no internet connection is required for core functionality. The GCC does not collect, transmit, or store personal or sensitive biological information, ensuring full compliance with data-protection and privacy standards.

The GCC was designed to support manual cell scoring in a variety of genotoxicity assays, with a focus on usability, portability, and data integrity. Users can define the number of events to be counted for each test directly within the interface, allowing standardization according to specific assay protocols. The interface features large, customizable buttons for increasing or decreasing cell counts, allowing users to adapt the layout to specific requirements using increment or decrement modes, respectively. Sessions can be saved and restored, enabling users to pause and resume counting without data loss, which is particularly useful during extended microscopy work or in field conditions (Figure 1).

**Figure 1 -:**
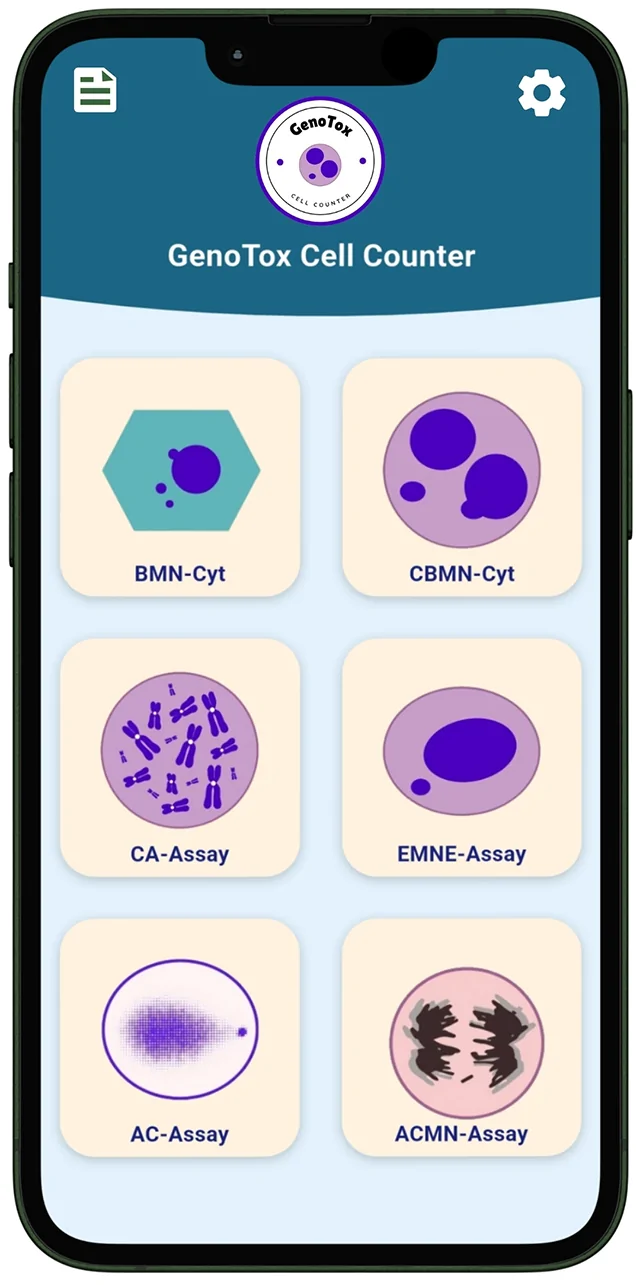
Main interface of the GenoTox Cell Counter (GCC) application, showing cell-counting screen, assay selection menu, and session-management options. Buccal MN Cytome Assay (BMN-Cyt), Cytokinesis-block MN Cytome Assay (CBMN-Cyt), Chromosomal Aberration Assay (CA-Assay), Erythrocyte Micronucleus Assay (EMNE-Assay), Alkaline Comet Assay (AC-Assay), MN in *Allium cepa* Assay (MNAC-Assay).

To facilitate data organization, the app includes category tagging, allowing counts to be labeled by assay type, including Buccal MN Cytome Assay (BMN-Cyt), Cytokinesis-block MN Cytome Assay (CBMN-Cyt), Alkaline Comet Assay (AC-Assay), MN in *A. cepa* Assay (MNAC-Assay), Erythrocyte Micronucleus Assay (EMNE-Assay), Chromosomal Aberration Assay (CA-Assay). Additionally, the app incorporates the specific cell types recommended by international guidelines for each assay, ensuring that all critical cellular structures are systematically recorded (Fenech et al., 1999; Thomas et al., 2009; Rodríguez-Romero et al., 2012; OECD, 2016; Canedo et al., 2021; Cardoso et al., 2022).

Results can be exported in structured formats such as TXT, CSV, or PDF, and shared via email or messaging platforms, streamlining collaboration and reporting. Error correction tools such as undo, reset, and confirmation prompts help maintain data accuracy throughout the scoring process. In addition, the application provides options to delete and rename tests individually, as well as delete all files at once or share them collectively. The user interface was optimized for clarity and minimal cognitive load, with visual feedback, low text input requirements, compatibility with smartphones, and multi-language support to ensure broad accessibility. These design choices were guided by the practical needs of toxicology researchers working in laboratory and field environments, ensuring that the app remains intuitive, reliable, and adaptable across diverse experimental protocols.

Two researchers (GPP and YA) initially performed paired manual cell counts on a representative subset of slides from various genotoxicity assays, including BMN-Cyt and AC-Assay. Each slide was scored twice: once using the custom-built mobile application (installed on Android devices such as Samsung Galaxy A15 and M51) and once using traditional manual methods (paper tally sheets and mechanical counters).

To ensure consistency and facilitate comparative analysis, specific cellular structures were quantified for each assay type, including MN, binucleated (Bin) cells, and nuclear buds (NBUDs). For each slide, cellular observations were performed manually under optical microscopy, and the corresponding findings were recorded directly in the mobile application (Figure 2). The app-based counts were stored, exported, and reviewed for accuracy and reproducibility, demonstrating its utility as a portable and efficient alternative to conventional paper-based or mechanical counting methods.

**Figure 2 -:**
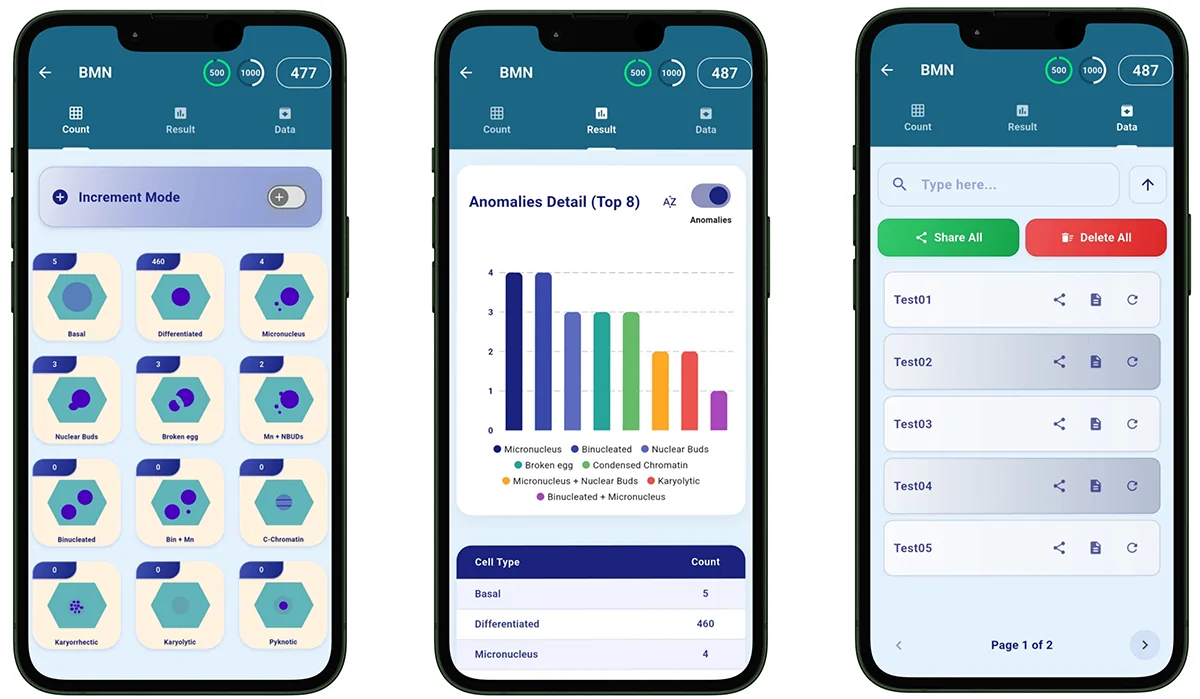
Screenshot of the active cell counting screen during a Buccal micronucleus (BMN-Cyt) assay. The interface displays customizable buttons for different cell types, along with their respective running tallies. Counts are entered in real time as the observer examines cells under optical microscopy, enabling accurate digital recording, organization, and traceability of genotoxicity data. Test: example of saved BMN-Cyt counts.

Importantly, the app replicates the logic and workflow of manual paper-based counting, offering the same analytical rigor while optimizing data entry, organization, and traceability through a digital interface.

Prior to its release on Google Play (https://play.google.com/store/apps/details?id=com.genotox.app) the app was initially tested by a group of 22 independent testers, including scientists and students. Over one year of continuous use in laboratory and academic settings, no software failures were reported, confirming the app’s stability and reliability under real-world conditions.

The fundamental goal of the GCC is not to automate microscopic analysis, but rather to modernize the manual counting workflow. This tool replaces traditional, error-prone tallying methods with a reliable digital system that preserves the researcher’s critical role in cell identification. This design philosophy is directly analogous to that of the Hematology Tally Counter app developed by Gupta et al. (2023), a validated tool that digitizes the manual differential counting process in hematology without attempting to replace expert analysis.

The validation of GCC yielded results in two primary areas: technical fidelity and usability. First, technical fidelity was confirmed through initial testing, which demonstrated that the tool accurately records user inputs, precisely replicating the logic of manual counting without introducing discrepancies. Its robustness was subsequently verified in a real-world laboratory setting over one year of continuous use, during which no software errors or data loss incidents were reported. This finding confirms the reliability of GCC for ensuring data integrity in research.

Second, a usability evaluation was conducted with a panel of 22 independent users, including scientists, students, and technicians. Qualitative analysis of their feedback revealed three primary strengths of the tool: 1) an intuitive user interface that minimizes the learning curve; 2) a session-saving feature that allows work to be securely paused and resumed; and 3) structured data export. These qualitative findings are consistent with quantitative validations of similar tools, such as the app by Gupta et al. (2023) which reported a high usability score (6.11/7 on the MAUQ) and was confirmed by users to be easy to use (95%).

In discussing these results, it is clear that the advantages of GCC over manual methods directly address common operational limitations. The tool’s technical reliability ensures immediate data traceability and the elimination of transcription errors, while its confirmed usability reduces cognitive load and optimizes time, particularly in high-throughput or educational settings. While GCC does not introduce a new analytical method, its contribution lies in modernizing a critical step in genotoxicity workflows. Its function is analogous to version control systems in software development: while it does not change the core work, GCC ensures that data records remain complete and traceable, thereby enhancing rigor and reproducibility in line with the principles of New Approach Methodologies (NAMs) in toxicology (Fortin et al., 2023).

Finally, the approach of GCC complements, rather than competes with, ongoing advances in AI-based cell detection, such as those reported by Jain and Khan (2023), who utilize deep learning models for cell detection. While tools like GCC strengthen and standardize manual workflows, AI-based approaches aim to automate specific analytical tasks. The value of GCC lies in its capacity to strengthen the current standard of manual scoring. Looking forward, it can also serve as a robust platform for validating emerging automated systems, thus creating a crucial bridge between human expertise and algorithmic precision.

The development and sustained use of the GCC demonstrate that targeted digital tools can significantly improve laboratory workflows while preserving established scientific practices. By emphasizing usability, portability, and structured data organization, GCC addresses operational challenges in manual cell scoring for genotoxicity assays. The app does not modify the analytical process but enhances the recording, review, and preservation of results, with digital tallies serving as verified, accurate sources for official documentation. Over one year of continuous use, no errors were reported, confirming the tool’s technical reliability. Its formal registration and public availability further support its broader implementation. GCC exemplifies how user-driven digital platforms can strengthen scientific rigor, improve efficiency, and promote reproducibility, serving both current research needs and as a foundation for validating emerging automated systems in genotoxicity testing. New features will continue to be added with each update, ensuring the application evolves alongside user needs and technological advances.

## Data Availability

 All relevant data supporting the findings of this study are included within the article.
